# Comparative Evaluation of Effect of Surface Roughness on Microbial Adhesion to Milled, 3D‐Printed, and Conventional PMMA Denture Base Resin: An In Vitro Study

**DOI:** 10.1155/ijod/8990537

**Published:** 2026-10-25

**Authors:** Jinsha Maji K., Sheejith M., Nivea Francis T.

**Affiliations:** ^1^ Department of Prosthodontics and Crown and Bridge KMCT Dental College Kozhikode Kerala India

**Keywords:** *Candida albicans*, denture base resin, microbial adhesion, polymethylmethacrylate, surface roughness

## Abstract

**Purpose:**

This study investigates how surface roughness influences *Candida albicans* adhesion to denture base resins fabricated using milling, 3D printing, and heat polymerization.

**Materials and Methods:**

Sixty polymethylmethacrylate (PMMA) disc specimens (10 mm diameter, 2 mm thickness) were prepared using three techniques: conventional heat‐cured PMMA, stereolithography (SLA)‐based 3D printing, and computer‐aided design and computer‐aided manufacturing (CAD‐CAM) milling (*n* = 20 per group). Surface roughness (Ra, µm) was recorded using a contact profilometer at three points per specimen. *C. albicans* adherence was evaluated by applying a standardized fungal suspension and counting colony‐forming units (CFUs) after 24 h of incubation. Specimens were fixed in 3% glutaraldehyde and dehydrated in graded ethanol. Data were expressed as mean ± standard deviation and analyzed via one‐way ANOVA, with significance set at *p* ≤ 0.05.

**Results:**

3D‐printed PMMA showed the highest roughness (Ra = 1.83 ± 0.428 µm), followed by conventional PMMA (Ra = 0.71 ± 0.111 µm), while milled PMMA had the lowest (Ra = 0.37 ± 0.060 µm). Differences were statistically significant (*p* = 0.001). Similarly, *C. albicans* adhesion was highest on 3D‐printed PMMA (1321.5 ± 271.05 CFU/mL), followed by conventional PMMA (903.75 ± 70.02 CFU/mL), and lowest on milled PMMA (372.7 ± 184.50 CFU/mL), with significant intergroup differences (*p* = 0.001). Post hoc LSD tests confirmed significance in all pairwise comparisons (*p* < 0.05). These results suggest smoother surfaces—particularly from CAD‐CAM milling—reduce microbial colonization.

**Conclusion:**

Fabrication method significantly influences *C. albicans* adhesion. CAD‐CAM milled PMMA demonstrated the least adherence due to its smoother surface, while 3D‐printed PMMA showed the highest due to surface irregularities. Surface roughness plays a crucial role, and CAD‐CAM milling appears most effective in minimizing fungal infection risk.

## 1. Introduction

Polymethylmethacrylate (PMMA) was discovered in the early 1930s by Rowland Hill and John Crawford [1]. PMMA resin is commonly used to fabricate dental prosthesis and implant‐supported denture due to good esthetics, ease of manipulation, and cost‐effectiveness. Acrylic resin has many inherent drawbacks, like water sorption, release of residual monomer, and also an unpolished intaglio surface promotes fungal adhesion, leading to denture stomatitis [2, 3].

Rapid development of digital technologies (computer‐aided subtractive and additive manufacturing) led to the expansion of their application in the production of polymer denture [3]. Computer‐aided design and computer‐aided manufacturing (CAD‐CAM) dentures are milled from prepolymerized PMMA billets which are polymerized under high temperatures and pressure. CAD‐CAM dentures are reported to be less porous, and consequently, less likely to harbor virulent microorganisms [4]. Additive manufacturing by 3D printing technologies is based on light‐induced polymerization of liquid resin monomers, forming solid objects layer by layer [2, 3].

The oral cavity of healthy individuals is colonized by commensal bacteria and yeast. In denture wearers, along with age, lack of appropriate denture hygiene, and balance of normal flora disrupted, leading to colonization and infection of oral mucosa [5]. *Candida albicans* is most prevalent pathogen responsible for denture stomatitis. The prevalence of denture stomatitis is reported to be 50%–70% among denture wearers. *Candida* can not only cause various local diseases but also systemic conditions like precancerous lesion and dysplasia of oral mucosa.

Surface roughness (Ra) is an important property of the denture base material that influences plaque, microbial adhesion, and, subsequently, denture stomatitis. A rough denture surface provides more area for microbial adhesion. Surface irregularities on the denture base provide shelter for the microorganisms and protect them from the shear forces associated with mechanical cleaning, making their removal difficult even when using antimicrobial agents [5].

Although several studies have evaluated the properties of conventionally processed, milled, and 3D‐printed PMMA denture base resins, there is limited information in the literature regarding the relationship between surface roughness and *C. albicans* adhesion among these different fabrication techniques. Therefore, the present study was conducted to evaluate and compare the effect of surface roughness on *C. albicans* adhesion to milled, 3D‐printed, and heat‐polymerized PMMA denture base resins.

## 2. Materials and Methods

### 2.1. Study Design

This in vitro comparative study was conducted in the Department of Prosthodontics at KMCT Dental College, the Genobio Research Center in Kottakkal, and the Department of Mechanical Engineering at the National Institute of Technology, Calicut. This was approved by the Institutional Ethics Committee/Institutional Review Board. The study aimed to evaluate the effect of surface roughness on microbial adhesion of *C. albicans* on milled (Bloomden, PMMA blanks), 3D‐printed (d‐TEC, 3D Accuprint TM), and heat‐polymerized PMMA denture base resin (DPI, Dental Products of India, Mumbai).

### 2.2. Sample Grouping

The study includes three groups—group 1 (heat‐polymerized PMMA), group 2 (3D‐printed PMMA), and group 3 (CAD‐CAM milled PMMA). Each group contains 20 specimens. Surface roughness and microbial adherence will be checked for all specimens in each group.

A total of 60 disc‐shaped specimens, each measuring 10 mm in diameter and 3 mm in height, were fabricated using three different techniques: heat‐polymerized PMMA, 3D printing, and CAD‐CAM milling [4]. All specimens were uniformly finished using silicon carbide abrasive paper (400, 600, and 800 grit) using an automatic grinder to ensure consistent surface smoothness across groups [3]. For the heat‐polymerized group, standardized wax patterns were first prepared using a silicone mold. These wax patterns were then processed into heat‐polymerized PMMA resin specimens using the conventional flasking, packing, and heat‐curing technique.

For the CAD‐CAM PMMA specimens, digital disc models were first designed and saved as STL files. These were milled from prepolymerized CAD‐CAM blocks using a precision cutting machine to achieve standard dimensions. The 3D‐printed specimens were produced using a stereolithography (SLA) printer, which cured the resin layer‐by‐layer with a laser for high precision. Following polishing, the specimens were immersed in distilled water for 6 days, with daily renewal of the water, to reduce the effect of residual monomers before testing [5].

### 2.3. Measurement of Surface Roughness

The surface roughness was measured by using a profilometer (Mitutoyo SJ‐410). The specimen was supported on a profilometer. The surface roughness value (Ra, μm) was determined at three spots (one at a central position and two at the margins) on each of the 10 specimens from each material using a profilometric contact surface measurement device. The mean value was calculated to obtain general surface characteristics of the specimens [5].

### 2.4. *C. albicans* Adherence Assay

*C. albicans* was used as the test organism in this study. The fungal strains were cultured on Sabouraud Dextrose Agar (SDA) plates and incubated at 37°C for 48 h. A fungal suspension was then prepared from the cultured plates and standardized to a concentration of 1 × 10^8^ CFU/mL in sterile normal saline, matching the turbidity of the 0.5 McFarland standard [6]. A 100 µL aliquot of this suspension was placed on the surface of each specimen from the three groups and was incubated at 37°C for 1 h. Following incubation, the specimens were gently washed with normal saline for 5 s to remove nonadherent cells. The samples were then immersed in 1 mL of fresh culture media and shaken at 80 rpm for 30 min to facilitate the detachment of adherent microorganisms [7]. The resulting cell suspensions were collected, properly diluted, and plated on agar media. Colony‐forming units (CFUs) were counted after 24 h. Prior to these procedures, all specimens were fixed in 3% glutaraldehyde solution for 2 h and sequentially dehydrated in ethyl alcohol solutions with increasing concentrations of 50%, 70%, 90%, and 100% for 10 min at each stage [6].

### 2.5. Statistical Analysis

All statistical procedures were performed using the Statistical Package for Social Sciences (SPSS) Version 20.0. Calculations for the power of the study (80%) were conducted prior to the commencement of the study. All quantitative variables were expressed as the mean and standard deviation, while qualitative variables were expressed in percentages. A one‐way ANOVA test was used to determine the association between qualitative variables. A probability value of *p* < 0.05 was considered statistically significant.

## 3. Results

### 3.1. Surface Roughness

The 3D‐printed PMMA group exhibited the highest surface roughness (1.83 ± 0.428 µm), which was significantly higher than heat‐polymerized PMMA group (0.71 ± 0.111 µm, *p* = 0.0001) and the CAD‐CAM milled PMMA group (0.37 ± 0.060 µm, *p* = 0.0001). Surface roughness was measured using a profilometer, with readings taken at three spots—one at the central position and two at the margins—on each specimen. The increased roughness in the 3D printed group was significantly higher compared to the other two groups (*p* < 0.05). An increased surface roughness is typically associated with higher susceptibility to microbial adherence and reduced polishability.

### 3.2. Microbial Adherence

Fabrication technique significantly affected *C. albicans* adherence (*p* ≤ 0.05). The 3D‐printed PMMA group demonstrated the highest mean adherence (1321.5 ± 271.05 CFU/mL), significantly surpassing the heat‐polymerized PMMA group (903.75 ± 70.02 CFU/mL) and the CAD‐CAM milled PMMA group (372.7 ± 184.50 CFU/mL). Pairwise comparisons revealed statistically significant differences between the 3D printed group and both the heat‐polymerized (*p* < 0.0001) and CAD‐CAM groups (*p* < 0.0001), as well as between the conventional and CAD‐CAM groups (*p* < 0.0001) (Table 1). These findings suggest that increased surface roughness in 3D‐printed PMMA may enhance microbial colonization.

**Table 1:** Multiple comparisons of surface roughness among heat‐polymerized, 3D‐printed, and CAD‐CAM milled PMMA denture base resins using the LSD post hoc test.

(I) groups	(J) groups	Mean difference (I−J)	Std. error	Sig.	95% confidence interval	
					Lower bound	Upper bound
Heat‐polymerized PMMA	3D printed	−1.12445^a^	0.08160	0.000	−1.2879	−0.9610
	CAD‐CAM milled PMMA	0.33885^a^	0.08160	0.000	0.1754	0.5023

3D‐printed PMMA	Heat cure	1.12445^a^	0.08160	0.000	0.9610	1.2879
	CAD‐CAM milled PMMA	1.46330^a^	0.08160	0.000	1.2999	1.6267

CAD‐CAM milled PMMA	Heat cure	−0.33885^a^	0.08160	0.000	−0.5023	−0.1754
	3D‐printed PMMA	−1.46330^a^	0.08160	0.000	−1.6267	−1.2999

^a^The mean difference is significant.

Table 2 demonstrates a statistically significant difference in surface roughness among the three PMMA fabrication techniques, as determined by one‐way ANOVA (*F* = 176.23, *p* = 0.001). The mean surface roughness values ranged from 0.37 to 1.83 µm, with the corresponding 95% confidence intervals ranging from 0.34–0.39 µm to 1.63–2.03 µm across the groups.

**Table 2:** Comparison of surface roughness among heat‐polymerized, 3D‐printed, and CAD‐CAM milled PMMA denture base resins.

Groups	*N*	Mean	Std. deviation	Std. error	95% confidence interval for mean		*F*‐value	*p*‐Value
					Lower bound	Upper bound		
Heat‐polymerized PMMA	20	0.71	0.111	0.024	0.65	0.76	176.23	0.001 ^∗^
3D‐printed PMMA	20	1.83	0.428	0.095	1.63	2.03		
CAD‐CAM milled PMMA	20	0.37	0.060	0.013	0.34	0.39		

*Note:* Statistically significant: one way ANOVA.

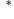
The mean difference is significant.

Table 3 demonstrates a statistically significant difference in *Candida* adherence among the three PMMA fabrication techniques (one‐way ANOVA, *F* = 120.67, *p* = 0.001). The mean values showed considerable variation among the groups, with the 95% confidence intervals reflecting the variability in *Candida* adherence within each fabrication technique.

**Table 3:** Comparison of *Candida* adherence among heat‐polymerized, 3D‐printed, and CAD‐CAM milled PMMA denture base resins.

Groups	*N*	Mean	Std. deviation	Std. error	95% confidence interval for mean		*F*‐value	*p*‐Value
					Lower bound	Upper bound		
Heat‐polymerized PMMA	20	903.75	70.02	15.65	870.97	936.52	120.67	0.001 ^∗^
3D‐printed PMMA	20	1321.5	271.05	60.60	1194.64	1448.35		
CAD‐CAM milled PMMA	20	372.7	184.50	41.25	286.39	459.10		

*Note:* Statistically significant: one way ANOVA.

^∗^The mean difference is significant.

Table 4 presents the LSD post hoc multiple comparison analysis for *Candida* adherence. Statistically significant differences were observed across all pairwise comparisons, including heat‐polymerized PMMA versus 3D‐printed PMMA (mean difference = −417.75, *p* < 0.001), heat‐polymerized PMMA versus CAD‐CAM milled PMMA (mean difference = 531.00, *p* < 0.001), and 3D‐printed PMMA versus CAD‐CAM milled PMMA (mean difference = 948.75, *p* < 0.001). The 95% confidence intervals for all comparisons excluded zero, confirming significant differences in *Candida* adherence among all three PMMA fabrication techniques.

**Table 4:** Multiple comparison of *Candida* adherence among heat‐polymerized, 3D‐printed, and CAD‐CAM milled PMMA denture base resins using the LSD post hoc test.

(I) groups	(J) groups	Mean difference (I−J)	Std. error	Sig.	95% confidence interval	
					Lower bound	Upper bound
Heat‐polymerized PMMA	3D printed	−417.75000^a^	61.21447	0.000	−540.3299	−295.1701
	CAD‐CAM milled PMMA	531.00000^a^	61.21447	0.000	408.4201	653.5799

3D‐printed PMMA	Heat cure	417.75000^a^	61.21447	0.000	295.1701	540.3299
	CAD‐CAM milled PMMA	948.75000^a^	61.21447	0.000	826.1701	1071.3299

CAD‐CAM milled PMMA	Heat cure	−531.00000^a^	61.21447	0.000	−653.5799	−408.4201
	3D‐printed PMMA	−948.75000^a^	61.21447	0.000	−1071.3299	−826.1701

^a^The mean difference is significant at the 0.05 level.

## 4. Discussion

The present study demonstrated statistically significant differences in both surface roughness and *C. albicans* adhesion among conventional heat‐polymerized, 3D‐printed, and CAD‐CAM milled PMMA denture base resins. Therefore, the null hypothesis was rejected. Among the three groups, CAD‐CAM milled PMMA exhibited the lowest surface roughness and the lowest *C. albicans* adhesion, whereas 3D‐printed PMMA demonstrated the highest values for both parameters. These findings indicate that the fabrication technique significantly influences the surface characteristics of PMMA and, consequently, its susceptibility to microbial adhesion.

The significantly lower surface roughness observed in the CAD‐CAM milled group may be related to the subtractive nature of the milling process and the characteristics of the material from which the specimens were fabricated. CAD‐CAM denture bases are milled from prepolymerized PMMA blocks or pucks manufactured under controlled conditions. Unlike conventional PMMA, which undergoes polymerization during denture fabrication, the milled specimens are produced from material that has already undergone polymerization [6]. The controlled production of these prepolymerized blocks may provide a more homogeneous material by minimizing processing‐related inconsistencies, while computer‐controlled milling allows standardized fabrication of the specimen geometry. These characteristics may explain the substantially lower surface roughness observed in the CAD‐CAM specimens compared with both conventional and 3D‐printed PMMA in the present study [6, 7].

The lower *C. albicans* adhesion observed in the CAD‐CAM milled group is consistent with its smoother surface. Radford et al. [7] reported greater adhesion of *C. albicans* to rough denture‐base surfaces than to smooth surfaces. Similarly, Al‐Dwairi et al. [8] reported superior surface roughness characteristics for milled PMMA compared with conventionally heat‐polymerized PMMA. The present findings are in agreement with these observations, as the CAD‐CAM milled group exhibited both the lowest surface roughness (0.37 ± 0.060 µm) and the lowest microbial adhesion (372.7 ± 184.50 CFU/mL). The reduced *C. albicans* adhesion observed with CAD‐CAM milled PMMA may therefore be attributed, at least in part, to its smoother surface, which provides fewer irregularities capable of facilitating microbial retention [9].

In contrast, the conventional heat‐polymerized group demonstrated intermediate surface roughness (0.71 ± 0.111 µm) and *C. albicans* adhesion (903.75 ± 70.02 CFU/mL). This finding may be explained by the inherent variability associated with conventional processing. During conventional denture fabrication, polymerization occurs within the mold after the polymer and monomer are mixed and packed [10]. Variations during mixing, packing, compression, and polymerization may contribute to surface irregularities and internal or surface porosities. Factors such as air entrapment, inadequate polymer‐monomer mixing, residual monomer, and insufficient compression have been proposed as potential contributors to porosity in conventionally processed acrylic resin [6]. Such irregularities may increase the available surface area and provide protected sites that facilitate microbial retention. Therefore, the higher roughness and *C. albicans* adhesion observed in the conventional group compared with the CAD‐CAM milled group may reflect the greater potential for processing‐related surface imperfections [10].

The highest surface roughness (1.83 ± 0.428 µm) and *C. albicans* adhesion (1321.5 ± 271.05 CFU/mL) were observed in the 3D‐printed group. These findings are consistent with the observations of Meirowitz et al. [9], who reported greater *C. albicans* adhesion to 3D‐printed denture base materials than to heat‐cured and milled materials. Similarly, Osman et al. [11] found that 3D‐printed specimens exhibited the greatest surface roughness and the highest *C. albicans* adhesion and biofilm formation among conventional, CAD‐CAM milled, and 3D‐printed denture base materials. Thus, the present findings support previous evidence demonstrating that 3D‐printed denture base materials may exhibit greater surface roughness and microbial adhesion than conventionally processed and milled materials.

The higher surface roughness observed in the 3D‐printed group may be explained by the additive, layer‐by‐layer nature of the printing process. Unlike milling, in which material is removed from a prepolymerized block to produce the desired geometry, 3D printing builds the specimen incrementally by depositing and polymerizing successive layers of resin [11]. The interfaces and boundaries between these layers can produce microscopic steps, ridges, and surface irregularities. These features may remain on the final surface even after postprocessing and can increase the effective surface area available for microbial retention. In the present study, this construction mechanism provides a plausible explanation for the substantially greater surface roughness observed in the 3D‐printed specimens compared with the milled specimens [12].

The influence of the printing technique on the surface morphology has also been demonstrated in studies comparing different additive manufacturing technologies. SLA and digital light processing (DLP) can produce different surface textures, with SLA specimens reported to exhibit more irregular surfaces than DLP specimens [12, 13]. However, Li et al. [14] reported no significant difference in *C. albicans* adhesion between SLA‐ and DLP‐fabricated resins. This suggests that although the specific printing technology may influence surface topography, microbial adhesion is likely determined by the combined effect of several material and surface characteristics rather than by the printing technology alone. Therefore, the greater *C. albicans* adhesion observed in the 3D‐printed group in the present study should be interpreted in relation to its measured surface roughness and the characteristics of additive manufacturing rather than attributed solely to the use of 3D printing [15].

The present findings are further supported by the study comparing conventional, CAD‐CAM milled, and 3D‐printed denture base resins, in which the higher microbial adhesion observed in 3D‐printed specimens was attributed to surface irregularities produced by the layer‐by‐layer manufacturing process [15]. The agreement between those findings and the present results strengthens the interpretation that additive manufacturing can generate a surface topography more conducive to *C. albicans* retention than the surface produced by CAD‐CAM milling under the conditions evaluated.

Although the differences in microbial adhesion closely followed the differences in surface roughness in the present study, surface roughness should not be considered the sole determinant of *C. albicans* adhesion [6]. Surface irregularities can provide niches for microorganisms and may reduce the effectiveness of mechanical cleaning; however, material composition, surface energy, hydrophobicity, residual monomer content, porosity, and other surface characteristics may also contribute to microbial attachment. Therefore, the present findings support surface roughness as an important contributing factor while recognizing that the observed differences between fabrication techniques may result from the combined effects of processing and material characteristics [10].

The present findings have potential clinical relevance because denture surfaces that facilitate microbial retention may contribute to the development and persistence of denture‐associated fungal infections. The substantially lower *C. albicans* adhesion observed on CAD‐CAM milled PMMA suggests that the smoother surface generated by this fabrication technique may provide an advantage in reducing microbial retention compared with conventional and 3D‐printed PMMA. However, this finding should not be interpreted as evidence that CAD‐CAM milling eliminates the risk of microbial colonization. Rather, under the standardized in vitro conditions of the present study, CAD‐CAM milling produced a surface that was less conducive to *C. albicans* adhesion [16].

The present findings should be interpreted in light of the limitations of the study. The investigation was performed under controlled in vitro conditions and therefore did not reproduce the complex oral environment. Factors such as saliva, changes in pH and temperature, mechanical wear, denture cleansing procedures, and individual oral hygiene practices may influence microbial adhesion in vivo. In addition, the study evaluated short‐term *C. albicans* adhesion rather than long‐term biofilm development. The relatively limited sample size and the use of a standardized *C. albicans* strain may also restrict the generalizability of the findings. Furthermore, the present study did not evaluate the influence of different postprocessing protocols, surface treatments, material modifications, or antimicrobial coatings, all of which may alter surface characteristics and microbial adhesion [17].

Future studies should therefore investigate whether optimization of postprocessing and surface finishing can reduce the roughness of 3D‐printed denture bases and consequently decrease *C. albicans* adhesion [14]. Further in vitro and in vivo investigations using larger sample sizes and longer biofilm‐development periods are also warranted [18]. Comparative evaluation of different printing technologies, printing orientations, postprocessing procedures, and surface treatments may help determine whether the higher microbial adhesion observed with 3D‐printed PMMA can be minimized [14, 16].

Overall, the present study demonstrates that the fabrication technique significantly influences both surface roughness and *C. albicans* adhesion of PMMA denture base resins. CAD‐CAM milled PMMA exhibited the smoothest surface and the lowest microbial adhesion, whereas 3D‐printed PMMA exhibited the roughest surface and the highest microbial adhesion. The findings are consistent with previous evidence and provide plausible explanations based on the fundamental differences between subtractive, conventional polymerization, and additive manufacturing processes. The results therefore suggest that the smoother surface produced by CAD‐CAM milling may offer an advantage in limiting *C. albicans* retention compared with conventional and 3D‐printed PMMA under the conditions investigated.

## 5. Conclusion

Within the limitations of the study, it can be concluded that the fabrication technique of denture base resins significantly influences *C. albicans* adhesion. CAD‐CAM milled PMMA resins demonstrated the lowest level of *C. albicans* adhesion, whereas 3D printed PMMA resins exhibited the highest level of adhesion, which can be attributed to the differences in surface roughness observed among the fabrication techniques. CAD‐CAM milled PMMA exhibited the lowest surface roughness, whereas 3D‐printed PMMA exhibited the highest surface roughness, which was likely responsible for the corresponding differences in *C. albicans* adhesion. These findings suggest that surface roughness is an important factor influencing *C. albicans* adhesion to PMMA denture base resins.

## Author Contributions

**Jinsha Maji K.**: conceptualization, data curation, investigation, methodology, project administration, visualization, validation, writing – original draft resources, writing – review and editing. **Sheejith M.**: data curation, investigation, methodology, visualization, writing – original draft resources, writing – review and editing. **Nivea Francis T.**: methodology, investigation, writing – review and editing.

## Funding

No funding was received for this manuscript.

## Disclosure

All authors substantially contributed to the conception of the work, read, and approved the manuscript.

## Conflicts of Interest

The authors declare no conflicts of interest.

## Data Availability

The data that support the findings of this study are available upon request from the corresponding author. The data are not publicly available due to privacy or ethical restrictions.
